# Partial hydrolyzed protein as an alternative stabilizer for peanut (*Arachis hypogaea*) butter

**DOI:** 10.1016/j.fochx.2025.102671

**Published:** 2025-06-17

**Authors:** Saban Thongkong, Kanyasiri Rakairyatham, Pipat Tangjaidee, Kridsada Unban, Wannaporn Klangpetch, Yuthana Phimolsiripol, Pornchai Rachtanapun, Saroat Rawdkuen, Jaspreet Singh, Lovedeep Kaur, Utthapon Issara, Passakorn Kingwascharapong, Suphat Phongthai

**Affiliations:** a School of Agro-Industry, Faculty of Agro-Industry, Chiang Mai University, Chiang Mai 50100, Thailand; b Center of Excellence in Agro Bio-Circular-Green Industry (Agro BCG), Faculty of Agro-Industry, Chiang Mai University, Chiang Mai 50100, Thailand; c Unit of Innovative Food Packaging and Biomaterials School of Agro-Industry, Mae Fah Luang University, Chiang Rai 57100, Thailand; d School of Food and Advanced Technology, Massey University, Palmerston North 4442, New Zealand; e Division of Food Science and Technology Management, Faculty of Science and Technology, Rajamangala University of Technology Thanyaburi, Pathum Thani 12110, Thailand; f Department of Fishery Products, Faculty of Fisheries, Kasetsart University, Bangkok 10900, Thailand

**Keywords:** Protein hydrolysate, Emulsifier, Peanut butter, Lipid oxidation, Antioxidant peptides.

## Abstract

Peanut protein hydrolysates with varying degrees of hydrolysis (DH) were prepared by using Alcalase and Flavourzyme. The enzymatic hydrolysis highly influenced a transformation of protein secondary structures, particularly from β-sheet to β-turn structures (11–21 %). The DH impacted functional properties and anti-free radicals' activity of peanut protein hydrolysates. Flavourzyme-derived protein hydrolysate (FPH) with DH5% had the maximum potential as an emulsifier (54.50 ± 0.71 %, *p* < 0.05). The effectiveness of protein hydrolysates in preventing the oil separation and enhancing the oxidative stability of peanut butter was dependent on the type of enzyme and DH. The inclusion of partial hydrolyzed protein (DH5%) produced by the Alcalase (APH) substantially decreased the occurrence of oil separation; whereas FPH with DH5% significantly retarded increment of PV, TBARs, CD in peanut butter during the storage period (*p* < 0.05). This study indicated the possible use of partial hydrolyzed proteins as a stabilizer in peanut butter by slowing lipid oxidation and increasing oil entrapment.

## Highlights

• Impacts of DHs on secondary structures of peanut protein were extensively reported.
• Correlation between protein's structure and functional properties were revealed.
• Partial hydrolyzed peanut protein could enhance peanut butter's storage ability.

## 1 Introduction

Peanut butter, a spread derived from finely ground roasted peanuts, is a type of nut spread that is commonly eaten in households and extensively utilized as an ingredient in the food industry. Peanuts retain their high protein and unsaturated fatty acid (UFA) content after being processed into peanut butter, making them an affordable and excellent source of protein and lipids. Due to its high nutritive value, peanut butter-based ready-to-use therapeutic food (RUTF) has been implemented in the diet program to treat malnutrition among children in underdeveloped countries (Stephenson et al., 2023).

Natural peanut butter consists of plant cell fragments combined with oil obtained from the grinding of the seed or nut. When compared to commercially stabilized peanut butter, it has a softer consistency and is more fluid. However, peanut butter without a stabilizer suffers from oil separation issues, as well as the formation of a rigid layer of peanut particles at the container's bottom due to settling. Further degradation occurs when lipids in peanuts, especially roasted peanuts, undergo oxidation, which results in a shortened shelf life, decreased nutritional value, and the development of rancidity, making peanut butter unsuitable for consumption (Sarpong, 2024). Antioxidants easily address these issues by reducing lipid oxidation through multiple mechanisms, including singlet oxygen quenching, metal ion chelation, and radical scavenging. Nonetheless, the necessity and desire for natural food products among consumers have increased over time. Certain consumers consciously choose processed foods with natural additives or those without any additional ingredients (Cao & Miao, 2023).

The food industry has made extensive use of synthetic additives such as butylated hydroxyanisole (BHA), butylated hydroxytoluene (BHT), and tertbutyl hydroquinone (TBHQ) because of their potent antioxidant properties, accessibility, and affordability (Ospina-Quiroga et al., 2022). However, consuming them can have several negative side effects, including gastrointestinal problems, skin sensitivities, and even a higher risk of cancer if consumed over an extended period of time. Food manufacturers find it difficult to find beneficial natural additives to replace synthetic ones. It is because most natural additives are not as effective as synthetic ones (Singh et al., 2024).

The search for natural stabilizers is gaining prominence in the food industry due to consumer demand. In addition to protein's nutritional value, scientists have studied and reported on its functional properties. It contains both hydrophobic and hydrophilic groups; therefore, it can act as an emulsifier. However, the fold structure of a native protein encloses the emulsification component, which limits its functional capabilities. Researchers employed an enzymatic hydrolysis of proteins to reveal hidden hydrophobic groups and make molecules more flexible, indicating that they have a high affinity for the oil-water interface (Tumpanuvatr & Jittanit, 2024). As a result, proteins can form a layer at the oil-water interface, which improves emulsion stability (Isnaini et al., 2021). However, excessive hydrolysis reduces the ability to emulsify while increasing antioxidant properties such as metal chelation, free radical scavenging activity, and reducing power (Aluko, 2015; Phongthai & Rawdkuen, 2020). Previous studies have shown the effectiveness of protein hydrolysates in reducing lipid oxidation in fish oil-in-water emulsions (Ospina-Quiroga et al., 2022) and improving the textural characteristics and shelf-life of fish emulsion sausages (Zakaria & Sarbon, 2018). Nonetheless, plant-based emulsion systems like additive-free peanut butter remain in encountering quality issues, specifically oil separation and oxidative rancidity during storage. While numerous studies have explored the effects of protein hydrolysate concentration on emulsion stability, comparatively few have investigated the influence of the degree of hydrolysis—an important determinant of the hydrolysates' functional and antioxidative properties. This gap highlights the need for targeted research on the application of protein hydrolysates with optimized hydrolysis degrees in plant-based emulsions. We hypothesize that such incorporation could substantially improve the oxidative and physical stability of products like peanut butter during storage.

As no research has been done on how a controlled-hydrolysis degree peanut protein fraction affects the stability of peanut butter storage, the purpose of this study was to investigate the impact of peanut protein hydrolysates with varying degrees of hydrolysis (DH) prepared by two commercial enzymes, Alcalase and Flavourzyme, on peanut butter storage stability. The physicochemical features of peanut protein hydrolysate were investigated, including the degree of hydrolysis (DH), functional qualities, antioxidant properties, protein patterns, and secondary structural modifications. Furthermore, the quality deterioration of peanut butter, including oil separation and oxidative stability throughout a 5-week storage period, was examined.

## 2 Materials and methods

### 2.1 Materials

Peanuts (KAC431) were purchased from a local farm in Mae Hong Son Province, Thailand. Alcalase® enzyme from *Bacillus licheniformis* (2.99 Units/mL) was purchased from EMD Chemicals, Inc. (San Diego, CA, USA). Flavourzyme® enzyme from *Aspergillus oryzae* (500–1000 LAPU/g) was provided by Novozymes (Bagsværd, Denmark). DPPH (2,2-diphenyl-1-(2,4,6-tri nitrophenyl) hydrazyl), trolox ((±)-6-hydroxy-2,5,7,8-tetramethylchromane-2-carboxylic acid), and ABTS (2,2-azino-bis (3-ethylbenzothiazoline-6-sulfonic acid) diammonium salt) were purchased from Sigma-Aldrich (St.Louis, MO, USA).

### 2.2 Peanut protein sample preparation

Peanuts were deshelled and dried in a hot air oven at 70 °C for 16 h. Dry peanuts were pulverized, defatted with hexane (1:5) for 6 h, and then dried in a hot-air oven at 60 °C for 16 h. The defatted peanut powder was combined with distilled water (1,5), and the pH of the resulting slurry was corrected to 10 using 1 M NaOH. The extraction was carried out with a magnetic stirrer at 50 °C for 1.5 h. The mixture was centrifuged (Super T-21, Kendo Laboratory Products, San Francisco, CA, USA) at 5500*g* for 10 min, then the supernatant was collected. The pH of the solution was adjusted to 4.5 with 1 M HCl and centrifuged again. The protein precipitates were collected and suspended in distilled water and the pH of the slurry was adjusted to 7.0 using 1 M NaOH. In order to eliminate salts, the neutralized protein precipitate was further washed out three times with distilled water. Subsequently, the resultants were dried up in a freeze dryer. The resulting peanut protein powder had a purity of 77.76 ± 0.31 %, as analyzed by the Dumas method, using a nitrogen-to-protein con*v*ersion factor of 5.59. The extraction was performed in duplicate.

### 2.3 Peanut protein hydrolysate (pH) preparation

Peanut protein hydrolysis was performed using the pH-stat test, as described in our earlier study of Phongthai et al. (2020) with some modification. The solution of peanut protein and distilled water (3 %, *w*/*v*) was prepared, and then enzyme was added at the ratio of 100:1. Flavourzyme (pH 7.0, 50 °C) and Alcalase (pH 8.5, 55 °C) were used to prepare peanut protein hydrolysates (PHs) with DHs of 5 %, and 10 %, yielding hydrolysate fractions of FPH-DH5%, FPH-DH10%, APH-DH5%, and APH-DH10%, respectively. The pH of protein solution was maintained at the optimal pH of each enzyme by adding 0.1 M NaOH during hydrolysis. The enzyme activity was terminated in boiling water for 10 min, and then solution was centrifuged at 5500*g* at a temperature of 4 °C for 15 min. The supernatant was collected and pH-adjusted to 7.0 before freeze drying. The degree of hydrolysis (DH) was calculated using the following equation:

(1) $$\mathrm{DH}\;\left(\%\right)=\left({\mathrm{BN}}_{b}/{\mathrm{\alpha h}}_{\mathrm{tot}}M_{p}\right)\times 100$$

where B is the volume of NaOH used (mL), N_b_ is the normality of the NaOH, M_p_ is the mass of protein in grams (N × 4.60), h_tot_ is the total number of peptide bonds in the protein substrate, and α is the average degree of dissociation of the α-NH_2_ groups, which can be calculated from the following equation:

(2) $$\alpha =\left(10^{\mathrm{pH}-\mathrm{pKa}}/1+10^{\mathrm{pH}-\mathrm{pKa}}\right)$$

where pH is the optimal pH for the hydrolysis of the enzyme, and pK_a_ was calculated from the parameters of the conditions used.

### 2.4 SDS-PAGE pattern of peanut proteins

The SDS-PAGE patterns of peanut protein concentrate and hydrolysates were investigated using sodium dodecyl sulfate polyacrylamide gel electrophoresis (SDS-PAGE) following the method modified by Phongthai et al. (2020). The protein solutions were combined with a sample buffer containing 0.125 M Tris-HCl, pH 6.8, 4 % SDS, and 20 % glycerol in a 1:1 volume ratio. The protein markers and samples (15 μL) were placed onto 12 % separation gels and 4 % stacking gels. The electrophoresis apparatus was configured and operated with a consistent current of 15 mA each gel until the bromophenol blue marker had reached the bottom of the gel, approximately 45 min. The gels containing the samples were immersed in a solution of Coomassie Brilliant Blue R-250 and left to soak overnight. They were then treated with a solution of acetic acid and methanol while being gently agitated to remove excess stain. The molecular weight of proteins in unknown samples was determined by comparing their electrophoretic mobility with that of a known pre-stained protein marker (15–250 kDa) from Beijing Solarbio Technology Co. Ltd., China.

### 2.5 Determination of protein secondary structure changes

Secondary structural alterations of the protein samples were examined using a Fourier transform infrared spectrometer (FTIR) (Nicolet iS10, Thermo Fisher Scientific Co. Ltd., USA). Two grams of each sample were mixed with KBr and pressed into a pellet. A resolution of 4 cm^−1^ was used in the 400–4000 cm^−1^ range of the measurement. The analysis of spectra in the amide I region, ranging from 1600 to 1700 cm^−1^, was conducted using OriginPro 2022 software (OriginLab Corporation, Northampton, MA, USA). This software facilitated the separation of the spectra into multiple component peaks at specific wavenumbers: α-helices at 1657 cm^−1^, β-sheets at 1611 and 1626 cm^−1^, β-turns at 1673 and 1688 cm^−1^, and random coils at 1642 cm^−1^, as outlined by Liu et al. (2019). The OriginPro 2022 software was used for baseline adjustment, decon*v*olution, and second derivative fitting. A Gaussian model was selected for the separation of numerous components. Protein samples were analyzed to identify six peaks representing four components: α-helices, β-sheets, β-turns, and random coils. The areas of these peaks were used to calculate the portion percentage of each component.

### 2.6 Functional properties determination

#### 2.6.1 Emulsifying properties

The emulsifying properties including emulsifying activity (EA, %) and emulsion stability (ES, %) were determined as described by Phongthai et al. (2020). Protein solutions (5 % *w*/*v*, 10 mL) were mixed with an equal quantity of refined peanut oil and homogenized at 10,000 rpm for 1 min and then centrifuged at 1500*g* for 5 min. The emulsifying properties were calculated from the following equations:

(3) $$\text{Emulsifying activity}\;\left(\%\right)=\left(A/B\right)\times 100$$

(4) $$\text{Emulsion stability}\;\left(\%\right)=\left(A_{\text{incubate}}/B\right)\times 100$$

where A is the volume of the emulsified layer (mL) after centrifugation, B is the total volume (mL), and A_incubate_ is the emulsified layer (mL) after incubation at 80 °C for 10 min and centrifugation.

#### 2.6.2 Oil absorption capacity (OAC)

Peanut protein samples (0.5 g) were mixed with 5.0 mL soybean oil and centrifuged at 2000 *g* for 15 min. The pellets were allowed to drain for 30 min. The oil fraction was measured and calculated as the gram of soybean oil absorbed per gram of protein sample (g/g).

### 2.7 *In vitro* antioxidant activity

The scavenging activity of PC and PHs for radicals (DPPH and ABTS) was determined using a modified Phongthai et al. (2020) method. Protein samples (1 mg/mL, 0.5 mL) were reacted with 0.1 mM DPPH (2 mL) and then left to react in the dark for half an hour. The absorbance was measured at 517 nm using a spectrophotometer (GenesysTM 10S, Thermo Scientific, Waltham, MA, USA). The equation below was used to determine the percentage of inhibitory activity;

(5) $$\text{Inhibition activity}\;\left(\%\right)=\left[\left({\mathrm{Abs}}_{\text{control}}-{\mathrm{Abs}}_{\text{sample}}\right)/{\mathrm{Abs}}_{\text{control}}\right]\times 100$$

where Abs_control_ and Abs_sample_ are the absorbance of the control and sample, respectively.

The protein samples were determined for ABTS radical scavenging ability. Solutions of potassium persulfate (2.6 mM) and ABTS (7.4 mM) was mixed in a ratio of 1:1, then incubated for 12 h. The derived stock solution was diluted with DI water (1,60) to get a working solution. The protein solutions (150 μL) were mixed with a working solution (28.5 mL), then incubated for 2 h in the dark condition. The absorbance was measured at 734 nm. The sample blank was prepared by using methanol instead of DPPH and ABTS solution. The percentage of inhibition activity was estimated by using the same equation as to DPPH inhibition activity.

### 2.8 Peanut butter preparation

Approximately 150 g of peanut kernel were roasted for 60 min at 150 °C in a laboratory digital electrical oven (ELE-1450A, Heng Wei, Wuxi, JS, China). Forced air was used to chill the kernels before removing the skin. Afterwards, peanuts were finely processed for 30 min using a food processor (Kenwood FDP65.400 W, Kenwood Limited, UK). Peanut protein hydrolysates with varying degrees of hydrolysis were added to the mixture at concentrations ranging from 1 to 3 % (*w*/w). The mixture was constantly ground for 30 min to make the peanut butter samples.

### 2.9 Storage stability determinations

#### 2.9.1 Storage condition and sampling

Each peanut butter sample weighed 25 g and was sealed in a polypropylene container (3.5 cm height, 5.5 cm diameter) with 0.6 ± 0.1 cm of headspace. The peanut butter samples were kept at room temperature of 27 ± 2 °C and sampled at 0, 3, and 5 weeks for further testing.

#### 2.9.2 Oil separation

The oil separation was measured over 0, 3, and 5 weeks following the method of Gong et al. (2018), with slight changes. A peanut butter sample (10 *g*) was centrifuged at 1500*g* for 25 min, after which the oil separation was estimated using the following equation:

(6) $$\text{Oil separation}\;\left(\%\right)=\left[\text{Weight of oil separated}\;\left(g\right)/\text{Weight of peanut butter before separation}\;\left(g\right)\right]\times 100$$

#### 2.9.3 Oxidative stability

##### 2.9.3.1 Peroxide value (PV)

Modified method of the AOCS Official Method for small-scale sample (Crowe & White, 2001) was used to determine PV in oil separated from peanut butter. Briefly, an oil sample (0.5 g) was titrated with sodium thiosulfate (0.001 M) and the result was expressed as mol/g sample.

##### 2.9.3.2 Conjugated dienes (CD)

CD in oil separated from peanut butter was determined by dissolving sample in isooctane and measuring the absorbance at 234 nm (Rakariyatham et al., 2021). Fold increase of CD was expressed as a ratio of an increased CD number detected in products upon storage to the original CD number detected in freshly prepared products.

##### 2.9.3.3 Thiobarbituric acid reactive substances (TBARs)

The determination of TBARs in oil extracted from peanut butter was conducted using a spectrophotometric approach as described by John et al. (2005). A 50 μL sample of oil was measured and combined with a mixture containing 0.375 % thiobarbituric acid (TBA), 15 % trichloroacetic acid (TCA), and 0.25 M hydrochloric acid (3.5 mL). The solution was subjected to heating in a water bath at its boiling point for a duration of 10 min. Following the cooling procedure with water, centrifugation was conducted at a speed of 6000*g* for 10 min. The measurement of the supernatant's absorbance was taken at a wavelength of 532 nm. The value of TBARs was calculated using a standard curved made with malondialdehyde (MDA) and expressed as ug MDA equivalent per gram of oil.

### 2.10 Statistical analysis

The data were presented as mean ± SD (*n* ≥ 3). The results were analyzed using one-way ANOVA using the IBM SPSS Statistics version 17.0 software (SPSS Inc., Chicago, IL, USA). Duncan's multiple range comparison was used to determine significant differences at *p* < 0.05. In addition, protein hydrolysates fractions, functional properties, and secondary structures were used for principal component analysis (PCA).

## 3 Results and discussion

### 3.1 Hydrolysis profiles of peanut protein

**Fig. 1:**
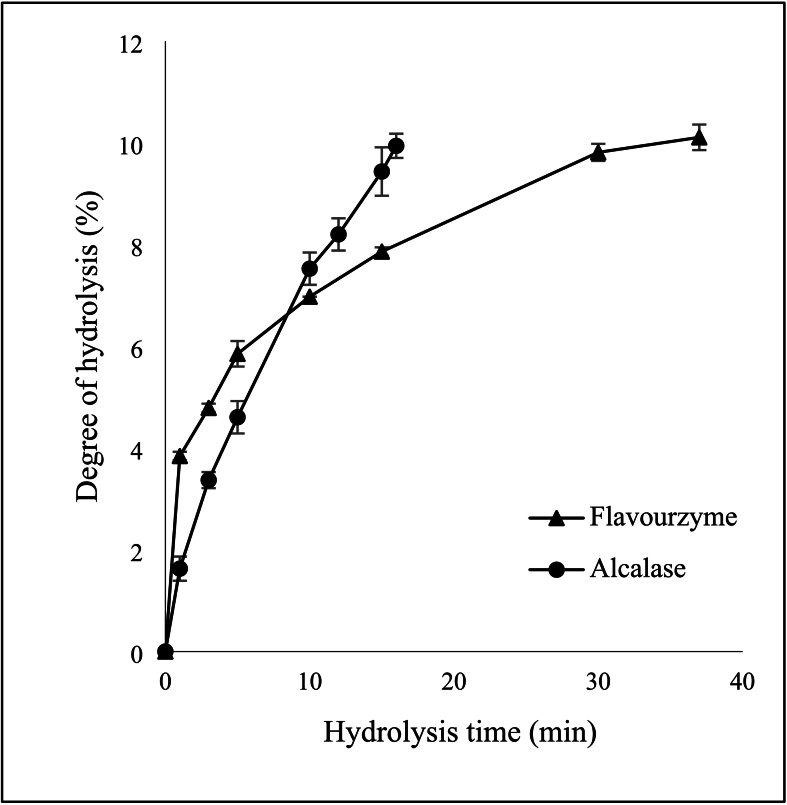
Effect of hydrolysis time on the degree of hydrolysis of peanut protein hydrolysates using Alcalase and Flavourzyme. Results are reported as mean ± SD. Error bar denotes standard deviation.

Peanut protein hydrolysates with different DHs were prepared using Alcalase and Flavourzyme. The protein hydrolysates prepared by Alcalase with DHs of 5 % (APH-DH5 %) and 10 % (APH-DH10 %) were obtained at the hydrolysis times of 4.7 and 15.7 min, respectively, while Flavourzyme hydrolyzed protein with DHs of 5 % (FPH-DH5%) and 10 % (FPH-DH10 %) were derived at 3.4 min and 36.5 min of hydrolysis time (Fig. 1), respectively. In the initial phase, the protein hydrolysis rate of Alcalase was similar to that of Flavourzyme. Nevertheless, the time required for Alcalase to attain a DH of 10% was significantly shorter. This is because of Alcalase, a serine *endo*-peptidase characterized by its broad substrate specificity, is capable of hydrolyzing peptide bonds within protein molecules, specifically those located at the carboxyl groups of the following amino acids: glutamic acid, methionine, leucine, tyrosine, and lysine (Phongthai et al., 2020; Siriwat et al., 2023). Our previous research Phongthai et al. (2020) supported this result by demonstrating that the same variety of peanut protein was a rich source of the aforementioned amino acids. Thus, it can be concluded that the peanut protein utilized in this study was a suitable substrate for Alcalase hydrolysis.

In contrast, Flavourzyme is an enzyme mixture derived from fungi, comprising partial *exo*- and endo-peptidases. Flavourzyme exhibited lower hydrolysis potential, especially when a degree of hydrolysis (DH) is higher than 6 %, compared to Alcalase at equivalent enzyme units over the same hydrolysis duration. This is because exo-proteases only function toward the ends of polypeptide chains, releasing a single amino acid residue, dipeptides, or tripeptides. The catalytic function of each enzyme type varies with substrate specificity that significantly influences the degree of hydrolysis. However, it was suggested that enzymatic hydrolysis using a combination protease can generate more efficient results than hydrolysis using a single enzyme (Kalita et al., 2024). In addition, increasing the ratio of enzyme and substrate protein can enhance the hydrolysis degree, thereby requiring a shorter hydrolysis duration.

### 3.2 SDS-PAGE pattern of peanut proteins

**Fig. 2:**
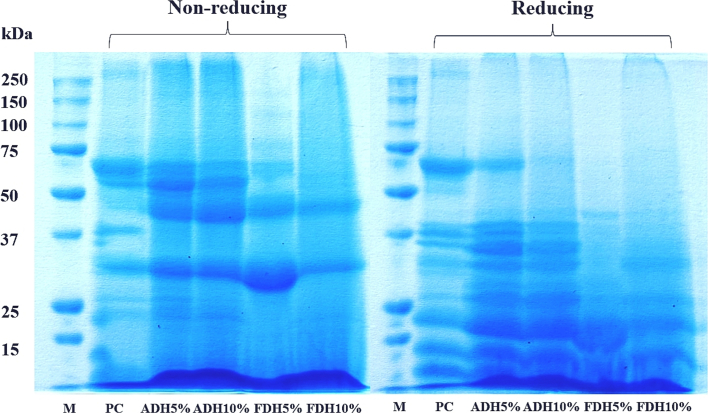
SDS-PAGE pattern of peanut proteins (M: protein marker, PC: protein concentrate, ADH5%: Alcalase-derived protein hydrolysate with DH5%, ADH10%: Alcalase-derived protein hydrolysate with DH10%, FDH5%; Flavourzyme-derived protein hydrolysate with DH5%, FDH10%; Flavourzyme-derived protein hydrolysate with DH10%).

The molecular weight distribution of the peanut protein concentrate and hydrolysates prepared by Alcalase and Flavourzyme at DH5% and 10 % are shown in Fig. 2. SDS-PAGE was used to separate proteins based on their molecular weight (MW) distribution under non-reducing and reducing conditions. Under the non-reducing condition, major bands found in the non-hydrolyzed protein had MWs ranging from 25 to 75 kDa. Following hydrolysis, an increase in DH from 5 to 10 % led to the disappearance of the protein bands at 50–75 kDa, and the observation of new bands at 32.5 and 46.6 kDa in both enzymes. This is because Alcalase and Flavourzyme hydrolysis obviously disrupted the protein chains, changing them into smaller molecules (Guo et al., 2022). Meanwhile, the protein pattern showed a slightly noticeable difference between non-reducing and reducing conditions. The protein band with MW of 46.6 kDa obviously disappeared under reducing conditions. This is because a strong reducing agent (β-mercaptoethanol) breaks down disulfide bonds (S—S) that are cross-linked between the -R group of cysteines in peanut protein polypeptide chains. It was also observed that some larger molecular weight bands disappeared after enzymatic hydrolysis, as evidenced by the appearance of new subunits at MWs of 13.4, 21.3, 29.0, 34.1, and 36.3 kDa in the hydrolysate fractions. These changes in protein molecular weight might have a positive impact on improving functional properties such as oil binding capacity, emulsification, and antioxidant activity (Guo et al., 2022; Phongthai & Rawdkuen, 2020; Siriwat et al., 2023).

### 3.3 Changes in protein secondary structures

**Fig. 3:**
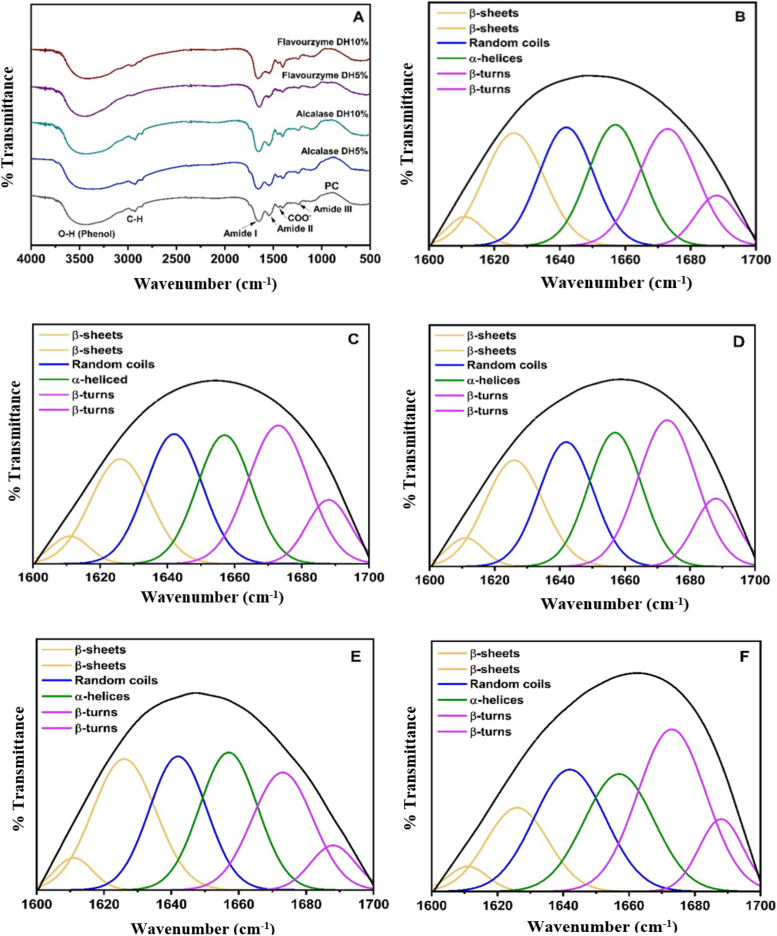
FTIR spectra (A) and peak-fitting of the secondary structure curves of peanut proteins; protein concentrate-PC (B), protein hydrolysates from Alcalase with DH5% (C) and DH10% (D), and protein hydrolysates from Flavourzyme with DH5% (E) and DH10% (F).

FTIR spectroscopy was employed to investigate the alterations in the secondary structures of peanut proteins subsequent to enzymatic hydrolysis, as illustrated in Fig. 3A. A Gaussian model was selected as the analytical framework for the multicomponent analysis. As stated by Alahmad et al. (2023), amide I is generally the most significant component for detecting alterations in the secondary structure of proteins, such as β-turns, β-sheets, and α-helices. The six peaks of each component in the protein hydrolysates are depicted in Fig. 3B-F. The average percentage of each secondary structure is provided in Table 1. Evidently, the native protein was composed of the following structures: 21.68 % random coil, 22.05 % α -helices, 26.30 % β-sheets, and 29.98 % β-turns. The structures of β-sheets and α-helices exhibited a marginal reduction in response to Alcalase hydrolysis at DH5% and 10 %, whereas the β-turn structures substantially increased in comparison to the protein concentrate (*p* < 0.05). Furthermore, the number of β-sheets, α-helices, and random coils increased significantly (p < 0.05) when Flavourzyme was used to degrade proteins at DH5%. Conversely, the number of β-turns decreased significantly (p < 0.05). The protein hydrolysate produced by Flavourzyme at DH10% exhibited a statistically significant reduction (p < 0.05) in the quantity of β-sheets and α-helices, but a substantial increase (p < 0.05) in the quantity of β-turns and random coils. As a result, enzyme type, hydrolysis degree, and amino acid compositions in proteins can all have an impact on protein secondary structure alteration. Furthermore, this finding was consistent with prior research that indicated enzymatic hydrolysis resulted in a significant loss of ordered protein structures in chickpea, soybean, and mung bean (Dent et al., 2023; Xie et al., 2019; Xu et al., 2020). These structural alterations have varying effects on the protein's functional properties. The α-helices and β-sheets secondary structures, along with their amphipathic abilities, are necessary for interfacial activity, resulting in good emulsifying ability (Ricardo et al., 2021). Zhang, Wang, et al. (2022) found that proteins with more α-helices and β-sheets have stronger structures, while those with more β-turns and random coils have more flexible structures.

**Table 1:** Secondary structure (portion, %) of peanut protein concentrate and hydrolysates.

Samples	Degree of hydrolysis (%)	β-sheets	β-turns	α-helices	Random coils
PC	0	26.30 ± 0.06 ^b^	29.98 ± 0.06 ^d^	22.05 ± 0.06 ^b^	21.68 ± 0.06 ^c^
APH	5	22.22 ± 0.05 ^c^	33.85 ± 0.07 ^c^	21.46 ± 0.06 ^c^	22.47 ± 0.06 ^b^
	10	22.16 ± 0.04 ^c^	35.17 ± 0.09 ^b^	21.96 ± 0.06 ^b^	20.72 ± 0.06 ^d^
FPH	5	27.66 ± 0.06 ^a^	26.71 ± 0.06 ^e^	23.29 ± 0.06 ^a^	22.34 ± 0.06 ^b^
	10	16.53 ± 0.05 ^d^	38.16 ± 0.10 ^a^	21.98 ± 0.08 ^b^	23.33 ± 0.08 ^a^

The mean ± standard error values with superscripts show significant differences (*p* < 0.05). PC: protein concentrate, APH: Alcalase-derived protein hydrolysate, FPH; Flavourzyme-derived protein hydrolysate.

### 3.4 Functional and antioxidant properties

#### 3.4.1 Oil absorption capacity

**Fig. 4:**
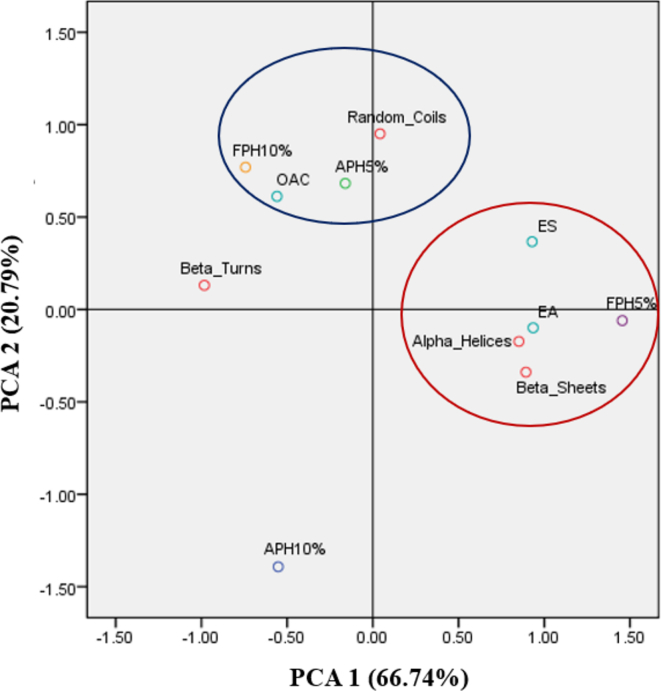
Principal Component Analysis (PCA) of protein hydrolysate fractions, functional properties, and secondary structures. FPH5%: Flavourzyme-derived protein hydrolysate with DH5%; FPH10%: Flavourzyme-derived protein hydrolysate with DH10%; APH5%: Alcalase-derived protein hydrolysate with DH5%; APH10%: Alcalase-derived protein hydrolysate with DH10%. OAC, EA, and ES refer to oil absorption capacity, emulsifying ability, and emulsion stability, respectively.

The oil absorption capacities of the peanut protein concentrates and hydrolysates are presented in Table 2. Enzymatic hydrolysis plays a key role in improving the oil absorption capacity of peanut protein. As a result, the protein concentrate had the lowest oil absorption capacity (0.51 ± 0.01 g oil/g sample), while APH-DH5% had the highest capability for oil absorption (0.81 ± 0.03 g oil/g sample, *p* < 0.05). However, the higher hydrolysis degree affected the reduction of this capacity of Alcalase-protein hydrolysates by about 27 %. On the other hand, the oil absorption capacity of FPH-DH10% (0.69 ± 0.08 g oil/g sample) was greater than that of hydrolysate with DH5% (0.52 ± 0.18 g oil/g sample). The higher oil absorption capacity might be because of the non-polar side chains that are exposed in protein hydrolysates and can bind to oil's hydrocarbon moieties. Also, the unfolded protein structure showing more hydrophobic groups can help physically trap oil, which also raises the oil absorption capacity (Russell et al., 2021). The principal component analysis (PCA) plot, depicted in Fig. 4, clearly corroborated these relationships. The plot offered an in-depth analysis of the correlations among several examined factors regarding protein functional properties and secondary structures. The analysis revealed that 87.53 % of the total variability in the data is noteworthy. The result showed that the random coils structure was found to be highly related to the oil absorption capacity of proteins. This finding was consistent with the investigation of Bergfreund et al. (2021) that the random-coil proteins had a strong ability to reduce the inter facial tension, resulting in a better interaction with oil phases.

**Table 2:** Functional and antioxidant properties of peanut protein concentrate and hydrolysates.

Samples	DH (%)	Oil absorption capacity (g oil /g sample)	Emulsifying ability (%)	Emulsifying stability (%)	DPPH radical scavenging activity (%)	ABTS radical scavenging activity (%)
PC	0	0.51 ± 0.01 ^c^	50.75 ± 2.47 ^b^	85.27 ± 2.07 ^b^	28.30 ± 3.31 ^c^	29.57 ± 1.50 ^d^
APH	5	0.81 ± 0.03 ^a^	48.00 ± 0.71 ^b^	91.16 ± 2.08 ^a^	79.57 ± 2.79 ^b^	57.02 ± 0.85 ^b^
	10	0.59 ± 0.07 ^c^	44.25 ± 1.06 ^c^	88.72 ± 1.33 ^a^	66.67 ± 2.18 ^c^	65.25 ± 1.72 ^a^
FPH	5	0.52 ± 0.18 ^c^	54.50 ± 0.71 ^a^	93.56 ± 2.68 ^a^	81.70 ± 1.12 ^b^	52.91 ± 2.60 ^c^
	10	0.69 ± 0.08 ^b^	39.25 ± 2.47 ^d^	89.90 ± 2.97 ^a^	87.45 ± 2.49 ^a^	62.13 ± 2.59 ^a^

The mean ± standard error values with superscripts show significant differences (*p* < 0.05). PC: protein concentrate, APH: Alcalase-derived protein hydrolysate, FPH; Flavourzyme-derived protein hydrolysate.

The oil absorption capacity of APH-DH10% (0.59 ± 0.07 g oil/g sample) and FPH-DH5% (0.52 ± 0.18 g oil/g sample) did not differ significantly from the non-hydrolyzed protein (*p* < 0.05). One possible explanation for the APH-DH10%’s oil absorption capacity is that the smaller molecules produced by this *endo*-protease cannot hold the oil in their structures as well as larger molecules (Alahmad et al., 2023; Muhamyankaka et al., 2013). Similarly, Phongthai et al. (2016) found that the oil absorption capacity of rice bran protein hydrolysate generated with Alcalase reduced significantly when the degree of hydrolysis was increased to 15.04 %. Meanwhile, low hydrolysis by Flavourzyme with DH5% may expose a significant portion of the protein's polar side chains and/or fold structure (Muhamyankaka et al., 2013), resulting in no difference in oil absorption capacity when compared to the protein concentrate.

#### 3.4.2 Emulsifying ability and emulsion stability

The emulsifying properties of the peanut protein concentrate and its hydrolysates are presented in Table 2. The emulsifying abilities of APH-DH5%, APH-DH10%, and FPH-DH10% were 48.00 ± 0.71 %, 44.25 ± 1.06 %, and 39.25 ± 2.47 %, respectively—significantly lower than that of the protein concentrate (50.75 ± 2.47 %) (*p* < 0.05). Meanwhile, FPH-DH5% (54.50 ± 0.71 %) was better in emulsifying the oil droplets, even compared to the unhydrolyzed protein. This result demonstrated a strong correlation between the emulsifying ability and the secondary structures of the protein (Fig. 4). FPH-DH5% had the most α-helices and β-sheet secondary structures, which are needed for good emulsifying and interfacial activity (Ricardo et al., 2021). In an alpha-helix, alkyl chain residues, notably in amino acids like alanine, leucine, and valine, typically orient outward from the helix core and do not participate directly in the hydrogen bonds that stabilize the helix. As a result, they may be attracted to the alkyl chain of oil and incorporated into the oil molecule, thereby improving emulsifying ability (Zhang, Fan, et al., 2022). Meanwhile, in a β-sheets, alkyl chain residues that are oriented outside of the sheet may also influence emulsion ability by enhancing hydrophobic interactions with oil molecules. These potential mechanisms may establish a stable interface around the oil droplets, reduce coalescence, and improve overall emulsification capability.

Regarding emulsion stability, protein hydrolysates prepared by Alcalase and Flavourzyme exhibited significantly superior performance compared to protein concentrate (*p* < 0.05). The emulsifying stability of APH-DH5%, APH-DH10%, FPH-DH5%, and FPH-DH10% was 91.16 ± 2.08 %, 88.72 ± 1.33 %, 93.56 ± 2.68 %, and 89.90 ± 2.97 %, respectively, substantially above that of the protein concentrate (85.27 ± 2.07 %) by approximately 3.45–8.29 % (*p* < 0.05). Protein structure flexibility may have a significant impact on the process of emulsification. The PCA plot demonstrates that α-helices and β-sheet structures, as indicated in Fig. 4, is highly associated with the emulsion stability of FPH-DH5%, implying a protein structure that is more adaptable and flexible (Zhang, Wang, et al., 2022). Additionally, the presence of exposed protein structures can enhance the formation of a strong protein-oil interface (Sun et al., 2023). Meanwhile, β-turn structure of peanut protein showed a negative correlation with emulsifying properties. However, hydrolysates with a higher DH had a lower capacity to stabilize emulsions. This is possibly due to low molecular weight peptides may not be sufficiently amphiphilic to exhibit effective unfolding and reorienting at the interface like large peptides, which contributes to the stability of emulsions.

#### 3.4.3 DPPH and ABTS radicals scavenging activity

The DPPH radical scavenging activities of Alcalase- and Flavourzyme-protein hydrolysates with different levels of hydrolysis degree are shown in Table 2. The protein hydrolysates exhibited more DPPH radical-scavenging action compared to the protein concentrate. It was also observed that the DPPH radical scavenging activity of the hydrolysates increased approximately 2–3 folds compared to the protein concentrate. Among protein hydrolysates, FPH-DH10% exhibited the maximum activity at 87.45 % (*p* < 0.05), while APH-DH10% demonstrated the lowest activity (66.67 ± 2.18 %, p < 0.05). Remarkably, the trends aligned with the oil absorption capability indicated in Table 2. Flavourzyme, a combination of *exo*- and endopeptidases, is more effective than Alcalase in enhancing the surface hydrophobicity of protein molecules during enzymatic hydrolysis. The hydrophobic DPPH radical, well dissolved in organic solvents like methanol, reacts better with Flavourzyme-protein hydrolysates as a result. The study also yielded comparable findings when examining alternative protein sources with distinct amino acid compositions, such as cowpea and peanut protein hydrolysates (Phongthai et al., 2020; Segura-Campos et al., 2012).

The ABTS radical scavenging activity of all protein hydrolysates demonstrated an increase with a higher degree of hydrolysis (DH), exhibiting a 24–36 % enhancement in comparison to the unhydrolyzed peanut protein. The protein hydrolysate with maximum hydrolysis degree prepared by Alacalase and Flavouzyme exhibited the highest ABTS radical scavenging activity (65.25 ± 1.72 % and 62.13 ± 2.59 %, respectively, *p* < 0.05). It may be due to a very extensive hydrolysis by the proteases used in this study suitably transforming large protein molecules into small-molecular-weight peptides with carrying functional groups like aromatic moieties, that are primarily responsible for antioxidant activity (Islam et al., 2022). Thus, those generated small peptides/amino acids were able to act as an electron donor and further converts free radicals into the more stable products (Islam et al., 2022; Isnaini et al., 2021). However, the antioxidant activity of protein hydrolysates can also be influenced by the protease used and the hydrolysis conditions. Changes in the size, quantity, and composition of free amino acids and short peptides also impact their antioxidative activity (Thongkong et al., 2023; Xiang et al., 2023).

This enhanced antioxidant activity has important implications for stabilizing lipid and oil-containing products such as peanut butter. Lipid oxidation is a primary factor contributing to quality degradation in peanut butter, resulting in rancidity, undesirable tastes, and diminished shelf life. These protein hydrolysates may enhance product quality during storage by scavenging free radicals and suppressing oxidative processes.

### 3.5 Storage stability of peanut butter

#### 3.5.1 Oil separation

The oil separation of peanut butter samples containing protein concentrate and hydrolysates is presented in Table 3. During the 5-week storage period, the oil separation in all peanut butter increased significantly (*p* < 0.05). The control sample (without any added proteins) had the greatest amount of separated oil during the storage period (5.39–7.04 %, p < 0.05). However, the addition of protein concentrate and hydrolysates contributed to the reduction of the separated oil layer in peanut butters. Protein hydrolysates, specifically the APH-DH5% fractions at 1 % and 3 % addition levels (exhibiting 5.67 % and 4.54 % oil separation, respectively, at the fifth week of storage), and the FPH-DH10% fractions at 1 % and 3 % addition levels (demonstrating 5.39 % and 4.77 % oil separation, respectively), were more effective in retarding oil separation than the protein concentrate, which displayed 7.04 % oil separation. Furthermore, during storage, the oil separation of peanut butter fortified with APH-DH5% was significantly lower than that of APH-DH10% (p < 0.05). Physical entrapment of Alcalase-hydrolysate could potentially be a critical factor in preventing oil separation. Consequently, in order to stabilize oil phases, a film must be formed using a protein molecule that is both larger and more flexible, which is obtained through a lower degree of hydrolysis. The results of this study indicated a significant positive correlation with the oil absorption capacity (Table 2 and Fig. 4). Therefore, the ability of proteins to absorb oil could potentially be a critical characteristic in preventing the separation of oils within food emulsions.

**Table 3:** Oil separation (%) of protein-enriched peanut butters during 5 week-storage.

Samples	Degree of hydrolysis (%)	Addition level (%)	Storage weeks		
			0	3	5
Control	–	0	5.39 ± 0.11 ^aC^	6.83 ± 0.10 ^aB^	7.04 ± 0.06 ^aA^
PC	0	1	4.25 ± 0.03 ^bC^	5.95 ± 0.34 ^bcB^	6.26 ± 0.30 ^bA^
		3	3.97 ± 0.07 ^cC^	4.71 ± 0.08 ^eB^	5.50 ± 0.39 ^cdA^
APH	5	1	3.46 ± 0.05 ^dC^	4.38 ± 0.47 ^fB^	5.67 ± 0.21 ^cA^
		3	2.36 ± 0.06 ^eC^	3.41 ± 0.17 ^gB^	4.54 ± 0.05 ^fA^
	10	1	3.66 ± 0.16 ^cdC^	5.71 ± 0.07 ^cB^	6.44 ± 0.17 ^bA^
		3	3.87 ± 0.12 ^cC^	6.00 ± 0.07 ^bB^	6.46 ± 0.25 ^bA^
FPH	5	1	4.21 ± 0.09 ^bC^	5.83 ± 0.10 ^bB^	6.20 ± 0.15 ^bA^
		3	4.12 ± 0.11 ^bC^	5.07 ± 0.10 ^dB^	5.76 ± 0.15 ^cA^
	10	1	3.81 ± 0.06 ^cC^	4.67 ± 0.27 ^efB^	5.39 ± 0.12 ^dA^
		3	3.48 ± 0.14 ^dB^	4.43 ± 0.20 ^fA^	4.77 ± 0.15 ^eA^

The mean ± standard error values with various superscripts in both rows (lower case alphabet) and columns (upper case alphabet) show significant differences (p < 0.05). PC: protein concentrate, APH: Alcalase-derived protein hydrolysate, FPH; Flavourzyme-derived protein hydrolysate.

In addition, the enrichment of protein levels in peanut butter influenced the separation of oil layer. The 1 % addition level of protein may not be sufficient to improve the storability of peanut butter. Meanwhile, the 3 % addition level of APH-DH5 % and FPH-DH10 % obviously reduced the separated oil by about 40–50 % compared to the control sample. This is probably caused by the increased viscosity of the continuous phase, which reduces the rate of movement of oil droplets within the peanut butter (Shevkani et al., 2019).

#### 3.5.2 Peroxide value (PV)

According to ISIRI 5690, the peroxide value is the only parameter mentioned in relation to peanut butter oxidation. Despite the fact that oxidation pathways are generally understood, there are numerous uncertainties regarding oxidation in actual situations, including production and storage. As a result, research in this area is limited. In this study, two levels of peanut protein concentrates and hydrolysates derived from enzymatic hydrolysis (Alcalase and Flavourzyme) were added to peanut butter. PV in peanut butter oil was monitored as an indicator of primary lipid oxidation product formation. As demonstrated in Table 4, PV gradually increased when stored at room temperature. During the 5-week storage period, it was observed that protein hydrolysate particularly APH-DH5 % at a 1 % addition level (8.40–11.39 mol/g sample PVs) and APH-DH10 % at a 1 % level (4.64–8.89 mol/g sample PVs) exhibited higher peroxide values (PVs) compared to their respective 3 % addition levels, which showed lower PVs of 4.83–7.50 mol/g sample and 1.55–8.68 mol/g sample, respectively. Similarly, FPH-DH5% and FPH-DH10% at 1 % addition levels showed higher PVs (5.20–11.10 mol/g sample and 1.42–8.84 mol/g sample, respectively) than at 3 % addition levels (1.61–7.72 mol/g sample and 0.89–8.41 mol/g sample, respectively). Peanut butter containing hydrolysates treated with Alcalase and Flavorzyme exhibited diminished PVs, particularly when used in larger quantities (3 % of the product). The extent to which protein hydrolysates enhanced the oxidative stability of peanut butter through a reduction in PV was also proportional to the degree of hydrolysis. When 1 % protein hydrolysates were added to peanut butter, protein hydrolysates with a higher DH of 10% from both enzymes reduced PV in the product significantly (*p* < 0.05) compared to protein hydrolysates with DH of 5%, the protein concentrate, and the control. These results revealed the antioxidant properties of peanut protein concentrate and hydrolysates, which corresponded to the DPPH and ABTS radical scavenging properties listed in Table 2. For the same reason, it has been reported that rice protein isolates and hydrolysates have the capacity to reduce PV and decrease linseed oil oxidation at a temperature of 45 °C (Gomes & Kurozawa, 2021).

**Table 4:** PV, TBARs, CD of proteins-enriched peanut butter during 5 week-storage.

Samples	Degree of hydrolysis (%)	Addition level (%)	Storage weeks	PV (mol/g sample)	TBARs (μg MDA equivalent / g sample)	CD (Fold increase)
Control	–	0	0	ND	15.78 ± 1.32 ^d^	–
			3	10.53 ± 0.01 ^a^	16.90 ± 0.54 ^d^	2.63 ± 0.15 ^a^
			5	10.95 ± 1.17 ^a^	29.91 ± 1.02 ^a^	2.63 ± 0.18 ^a^
PC	0	1	0	ND	12.88 ± 0.88 ^e^	–
			3	8.04 ± 0.91 ^bc^	20.63 ± 1.96 ^c^	1.89 ± 0.27 ^c^
			5	9.67 ± 0.51 ^b^	29.32 ± 0.44 ^a^	2.58 ± 0.33 ^a^
		3	0	ND	14.71 ± 1.90 ^d^	–
			3	5.45 ± 0.77 ^d^	16.60 ± 2.91 ^d^	1.60 ± 0.10 ^d^
			5	7.70 ± 0.89 ^c^	24.78 ± 2.05 ^b^	2.69 ± 0.53 ^a^
APH	5	1	0	ND	13.52 ± 2.17 ^d^	–
			3	8.40 ± 0.10 ^c^	16.23 ± 2.10 ^d^	1.73 ± 0.02 ^d^
			5	11.39 ± 0.87 ^a^	25.18 ± 1.43 ^b^	1.88 ± 0.28 ^cd^
		3	0	ND	13.07 ± 1.26 ^de^	–
			3	4.83 ± 0.35 ^d^	20.53 ± 0.68 ^c^	1.36 ± 0.30 ^e^
			5	7.50 ± 1.35 ^c^	30.00 ± 2.04 ^a^	2.31 ± 0.15 ^b^
	10	1	0	ND	13.56 ± 0.77 ^e^	–
			3	4.64 ± 0.33 ^d^	19.22 ± 2.08 ^c^	1.86 ± 0.26 ^cd^
			5	8.89 ± 0.64 ^b^	30.20 ± 2.11 ^a^	2.26 ± 0.34 ^bc^
		3	0	ND	14.71 ± 1.76 ^de^	–
			3	1.55 ± 0.07 ^e^	17.04 ± 3.76 ^c^	0.90 ± 0.16 ^f^
			5	8.68 ± 1.26 ^b^	28.63 ± 0.38 ^b^	1.37 ± 0.08 ^e^
FPH	5	1	0	ND	13.02 ± 0.72 ^e^	–
			3	5.20 ± 0.48 ^d^	21.15 ± 0.42 ^c^	1.31 ± 0.23 ^e^
			5	11.12 ± 0.81 ^a^	21.64 ± 0.45 ^c^	1.66 ± 0.15 ^d^
		3	0	ND	13.59 ± 1.13 ^de^	–
			3	1.61 ± 0.21 ^e^	20.49 ± 4.23 ^c^	0.84 ± 0.21 ^f^
			5	7.72 ± 0.09 ^c^	22.18 ± 0.61 ^c^	1.37 ± 0.13 ^e^
	10	1	0	ND	14.02 ± 0.55 ^de^	–
			3	1.42 ± 0.04 ^e^	27.24 ± 2.38 ^b^	0.97 ± 0.25 ^f^
			5	8.84 ± 0.69 ^b^	29.13 ± 4.81 ^ab^	1.65 ± 0.09 ^d^
		3	0	ND	14.36 ± 0.31 ^de^	–
			3	0.89 ± 0.15 ^f^	26.43 ± 2.51 ^b^	0.79 ± 0.18 ^f^
			5	8.41 ± 0.06 ^b^	29.40 ± 1.06 ^ab^	1.87 ± 0.30 ^cd^

The mean ± standard error values with superscripts show significant differences (p < 0.05). PC: protein concentrate, APH: Alcalase-derived protein hydrolysate, FPH; Flavourzyme-derived protein hydrolysate.

#### 3.5.3 Conjugated diene (CD)

CD is an additional biomarker of lipid oxidation since it forms *via* radical resonance during both initiation and propagation, resulting in a double bond shift (Shahidi & Zhong, 2010). Peanut butter held at room temperature for 3 to 5 weeks increased the CD of the product's oil, as demonstrated in Table 4. Incorporating hydrolysates and peanut protein concentrates significantly reduced CD production (p < 0.05), aligned with the change of PV. Protein hydrolysates made with Alcalase and Flavourzyme lowered CD levels in the products in a dose-dependent manner. The higher the DH, the lower the CD. As a result, a protein hydrolysate with DH10% in peanut butter performed much better than one with DH5%. Throughout the 5-week storage period, it was noted that peanut butter with the APH-DH10% fraction at a 3 % addition level demonstrated a lesser increase in conjugated diene (CD) content (0.90–1.37-fold), suggesting superior control of lipid oxidation in comparison to the formulation containing the APH-DH5% fraction at the same level, which exhibited a greater CD increase of 1.36–2.31-fold. In a similar manner, the peanut hydrolysate containing the FPH-DH10% fraction at a 3 % addition level showed enhanced oxidative stability, with a CD increase ranging from 0.84 to 1.37-fold. In contrast, the APH-DH5% fraction displayed a more significant increase, ranging from 0.79 to 1.87-fold. The results indicate that increased levels of hydrolysis (DH10%) in both Alcalase and Flavourzyme hydrolysates are more effective in reducing the formation of primary oxidation products during storage. Protein hydrolysates demonstrated consistency in the *in vitro* test (Table 2) and food emulsion model. The acquired results demonstrated the superior antioxidant activity of the produced short peptides and amino acids, which could act as electron donors and further convert free radicals into more stable compounds. This successfully prevents the delocalization of free radicals which results in the creation of CDs. According to these findings, plant-derived protein hydrolysates might effectively improve the oxidation resistance of lipid-containing products. Similarly, Liu et al. (2019) reported that fava bean protein hydrolysates made O/W emulsions more resistant to oxidation by lowering CDs when stored at 7 °C.

#### 3.5.4 TBARs

TBARs were measured in peanut butter oil enhanced with various peanut protein fractions to determine the production of secondary lipid oxidation products. The amount of TBARs in the peanut butter oil from the control sample went up to 16.90 ± 0.54 and 29.91 ± 1.02 μg MDA equivalent/g sample, after being stored at room temperature for 3 and 5 weeks. The addition of 1 % protein concentrate did not affect to TBARs value; meanwhile adding 3 % peanut protein concentrate to the product remained TBARs at 24.78 ± 2.05 μg MDA equivalent/g sample after 5 weeks of storage (*p* < 0.05). Among the protein hydrolysates, FPH-DH5% demonstrated the greatest significant reduction in MDA formation in peanut butter samples over a 5-week storage period (21.64–22.18 μg MDA equivalent/g sample, p < 0.05) across all examined addition levels. These results were consistent with PV and CDs, indicating that peanut protein concentrate and hydrolysates considerably increased the oxidative stability of peanut butter. It is possible that the antioxidative peptides in the protein hydrolysate scavenge free radicals to partially prevent the formation of lipid hydroperoxide during the propagation stage. However, some generated lipid hydroperoxides may be further broken down into secondary compounds by prooxidant metals. According to the peptide's antioxidant capability, it could function as a metal chelator to disrupt the peroxide breakdown reaction into secondary products, thereby lowering the TBARs value. Yu et al. (2020) supported this finding by discovering that enzymatic hydrolysis increased the metal chelating activity of protease-treated peanuts. Furthermore, Zhao et al. (2012) found that rice dreg protein hydrolysates can effectively inhibit TBARs production in a variety of emulsion-based food systems.

However, there was no significant difference in TBAR production among peanut butter enriched with APH-DH5%, APH-DH10%, FPH-DH10%, and the control sample (*p* > 0.05). This is most likely owing to the loss of crucial peptide and protein structures that can bind to metal ions in a variety of ways, including ionic bond formation, water coordination, electron density sharing, and hydrogen bond coordination (Ashaolu et al., 2023).

## 4 Conclusion

In this study, enzymatic hydrolysis can extend the functional properties of proteins, especially their oil absorption capacity and emulsification. The peanut protein hydrolysates prepared by two commercial enzymes, including Alcalase and Flavourzyme, with limited degree of hydrolysis are applicable for improving the storage stability of peanut butter products. The 3 % inclusion of partially hydrolyzed protein (DH5%) prepared by Alcalase significantly reduced oil separation in peanut butter during the 5-week storage. Our findings suggested that the partially hydrolyzed peanut protein can be used as a natural stabilizer that delays oil separation and inhibits lipid oxidation in peanut butter. Although, the partially hydrolyzed proteins offer benefits in stabilizing peanut butter without any interference tastes; however, the presence of bitter substances that generated by excessive hydrolysis may negatively affect customer perception and adoption.

## CRediT authorship contribution statement

**Saban Thongkong:** Writing – original draft, Validation, Methodology, Formal analysis, Data curation. **Kanyasiri Rakairyatham:** Writing – original draft, Methodology, Data curation. **Pipat Tangjaidee:** Writing – review & editing, Investigation, Formal analysis. **Kridsada Unban:** Writing – review & editing, Investigation, Formal analysis. **Wannaporn Klangpetch:** Writing – review & editing, Funding acquisition. **Yuthana Phimolsiripol:** Writing – review & editing, Funding acquisition. **Pornchai Rachtanapun:** Writing – review & editing, Funding acquisition. **Saroat Rawdkuen:** Writing – review & editing, Funding acquisition. **Jaspreet Singh:** Writing – review & editing. **Lovedeep Kaur:** Writing – review & editing. **Utthapon Issara:** Writing – review & editing. **Passakorn Kingwascharapong:** Writing – review & editing. **Suphat Phongthai:** Writing – original draft, Validation, Supervision, Project administration, Methodology, Investigation, Funding acquisition, Data curation, Conceptualization.

## Declaration of competing interest

The authors declare that they have no known competing financial interests or personal relationships that could have appeared to influence the work reported in this paper.

## Data Availability

Data will be made available on request.
